# Jack-of-All-Trades “Supergene” Controls Butterfly Wing Pattern Diversity

**DOI:** 10.1371/journal.pbio.0040329

**Published:** 2006-09-26

**Authors:** Liza Gross

While studying the local flora and fauna of the Amazon jungle in the 1860s, Henry Walter Bates made a striking discovery. Butterflies inhabiting a particular geographic region sported the same wing patterns—even though they were unrelated species. Bates proposed that nonpoisonous species had mimicked the patterns of noxious species that predators avoided, thus gaining a selective advantage. (This adaptation is viewed as one-sided; if predators eat the foul-tasting “model” butterfly, they learn to shun all butterflies with that pattern. But if they sample a palatable mimic, they’re likely to stop avoiding butterflies with that pattern—until they relearn their lesson.)

Today, scientists can use the tools of genomics and genetics to investigate the mechanism of convergent evolution—the emergence of similar physical traits (or phenotypes) in unrelated species. Such empirical studies have provided insight into a longstanding controversy spawned by evolutionary theorists over the origin of mimicry: does it arise gradually through the accumulation of random mutations by selection or in “phenotypic leaps” through the constraining influence of shared developmental pathways with a bias toward a particular phenotype?

Recent molecular studies found evidence that regulation or recruitment of the same genes or gene variants may explain convergent evolution. In a new study, Mathieu Joron, Chris Jiggins, and colleagues took a different approach by investigating the molecular basis of both convergent and divergent phenotypes. The involvement of the same genomic loci in convergent phenotypes suggests that developmental constraints give rise to these shared phenotypes. The presence of a multitude of convergent and divergent phenotypes in the wing patterns of *Heliconius* butterflies allowed the researchers to test the possibility that mimetic convergence results from constraints in the regulation of butterfly color patterns.

The researchers worked with three species of *Heliconius* butterflies, including Müllerian mimics (all mimetic species are poisonous, to mutual benefit). Two species, *H. melpomene* and *H. erato*, are distantly related yet have identical wing patterns. Both species radiated into over 30 races (or subspecies) in parallel, with the two species (“co-mimics) displaying a single pattern locally. The third species, *H. numata*, is closely related to *H. melpomene* but has radically different wing patterns, with up to seven different variations in a single region. Each of these variations mimics a different species of another butterfly genus, *Melinaea*.

In *H. melpomene*, variation in white and yellow patterns is controlled by at least three genomic loci—*N*, *Yb*, and *Sb*—that are tightly linked, or inherited together. Red pattern elements are controlled by another linked loci pair—*B* and *D*. In *H. erato*, the *Cr* locus produces patterning effects similar to the interaction of *N*, *Yb*, and *Sb* in *H. melpomene*, while an unlinked locus, *D*, appears to control red pattern variation much like the *B–D* pair does in *H. melpomene*. In polymorphic *H. numata*, all the mimicked color patterns derive from a single locus, *P*, thought to be a “supergene” (a tightly linked cluster of individual genes).

**Figure:**
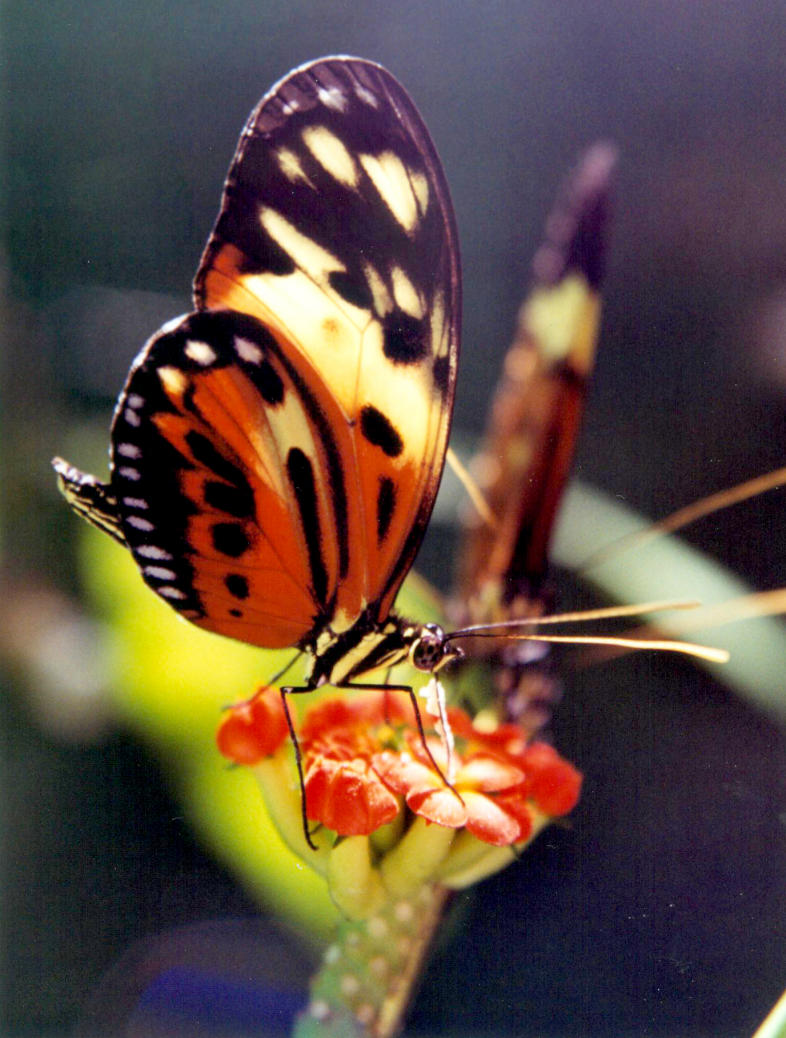
*Heliconius numata*, f. silvana feeding from a *Psiguria* flower. (Photo: Mathieu Joron)

The researchers crossed different races of each of the three species to explore the genetic basis of the variations. For example, two different subspecies of *H. melpomene* from different regions in Ecuador were crossed with an *H. melpomene* subspecies stock from French Guiana to produce second-generation offspring. Offspring were then genotyped to identify genes responsible for the resulting color patterns and to map the relevant major color-patterning loci—*N*, *Yb*, and *Sb* loci for *H. melpomene* crosses, *Cr* for *H. erato*, and *P* for *H. numata*—in individual offspring.

Using molecular markers developed in the region of the pattern genes, they found that the three loci controlling color pattern variation for each species inhabit the same genomic location. Indeed, the elements controlling white and yellow pattern variation in *H. melpomene* (*N*, *Yb*, and *Sb*) and *H. erato* *(Cr)* are tightly linked to genetic markers that occupy the same position in both species. Similarly, the locus *P*, which controls whole-wing variation in *H. numata*, is also linked to the same series of markers.

These results, Joron et al. conclude, suggest that a single conserved locus is responsible for producing wing pattern diversity in *Heliconius* butterflies. Rather than a constraining role, this locus provides what the researchers call a “jack-of-all-trades flexibility.” It presumably functions as a “developmental switching mechanism” for natural selection, they explain, by responding to a wide range of mimetic pressures to produce radically divergent, locally adapted wing patterns. Now researchers can begin to identify and determine the modus operandi of the genes at the center of what has been called a “developmental hotspot” to better understand how they drive the adaptive evolution of mimetic color pattern shifts. For more on the evolution of mimicry in butterflies, see the Primer (DOI: 10.1371/journal.pbio.0040341 in the October 2006 issue).

